# Social determinants and adherence to recommended COVID-19 vaccination among the Arab ethnic minority: A syndemics framework

**DOI:** 10.3389/fpubh.2022.1016372

**Published:** 2022-09-28

**Authors:** Anat Amit Aharon

**Affiliations:** School of Health Professions, Sackler Faculty of Medicine, Tel Aviv University, Tel Aviv, Israel

**Keywords:** COVID-19 vaccination, syndemics theory, acculturation style, health behavior, self-rated health status

## Abstract

**Background:**

Since the mass vaccination against SARS-CoV-2 was launched in Israel, the Arab ethnicity minority had lower vaccine uptake. The syndemics theory suggests a closely interrelated complex of health and social crises among vulnerable societies results in an increased disease burden or in more adverse health conditions. Syndemics may explain the health disparities between different people or communities. Likewise, acculturation was found to be associated with different health outcomes among minority populations. The purpose of the study is to explore the association between syndemic construct, acculturation style, and adherence to recommended COVID-19 vaccination among the Arab ethnicity in Israel.

**Methods:**

A cross-sectional study among 305 participants who completed a self-report questionnaire. Syndemic construct (syndemics score and syndemics severity) was calculated from the participants' health behavior index, self-rated health status, and adherence to flu vaccination. Four acculturation strategies were defined according to Barry's acculturation model: assimilation, integration, separation, and marginalization style. Linear regression (stepwise method) was conducted to determine the explanatory factors for COVID-19 vaccine adherence.

**Results:**

Assimilation and separation acculturation styles and syndemics severity were significantly associated with higher adherence to the recommended COVID-19 vaccination (*B* = 1.12, 95%CI = 0.34–1.98; *B* = 0.45, 95%CI = 0.10–0.80; B = 0.18, 95%CI = 0.09–0.28; respectively). The explained variance of the model (*R*^2^) was 19.9%.

**Conclusion:**

Syndemics severity, assimilation and separation acculturation styles were associated with higher adherence to recommended COVID-19 vaccination in the Israeli Arab minority population. Syndemics score was not associated with recommended COVID-19 vaccination. To encourage COVID-19 vaccination among minority communities, campaigns should be tailored to the social determinants in a sensitive and individualized manner.

## Introduction

In Israel, the mass vaccination against SARS-CoV-2 that caused the COVID-19 pandemic was launched in December 2020. As in other countries, the common vaccine in Israel is the mRNA based BNT162b2, a newly developed vaccine technology. The Health Maintenance Organization, HMOs, were in charge of implementing the vaccination process. According to the National Health Insurance Law, 1995 (1), all citizens of Israel have health insurance in one of four HMOs according to their choice. HMOs operate primary community health clinics throughout the country. However, there are accessibility disparities between various parts of the Arab minority and the Jewish population, for example, among the Bedouin who live in southern Israel (2, 3).

The population in Israel has diverse ethnic and religious sub-groups. Approximately 74% of the population are Jews and about 21% are Arabs (4). Throughout the vaccination process, there was a gap in the vaccination rate between the Jewish and the Arab minority populations. Namely, the Arab population had a slower and lower vaccine uptake of the first and second doses of the vaccine (5). For example, at the beginning of April 2021, the vaccination rate of the first dose in the Arab population was 38%, compared to the general Jewish population's rate of 57% (6). In general, the Arab population in Israel is characterized by a lower socio-economic status (SES) compared to the Jewish population (7). An analysis of the COVID-19 epidemic in Israel found a lower vaccination uptake in low SES cities, most of them Arab cities, compared to high SES cities in the general Jewish population (5). These findings have been confirmed in another study (8). Previous international studies have also found health disparities in morbidity, mortality and health outcomes due to COVID-19 between different populations with different SES's. Musey et al. (9) reported a 1.99 (95%CI = 1.92–2.05) higher risk of hospitalization and a 1.6 (95%CI = 1.50–1.71) higher mortality risk among black patients with COVID-19 compared to white patients with COVID-19 in the USA (9). This SES disparities pattern was additionally found in others countries like Japan (10), England (11) and Brazil (12). These studies showed that communities and populations with low SES are more vulnerable to illness including COVID-19 and its consequences. Indeed, socioeconomic determinants were found to be associated with an intention to vaccinate against COVID-19 or adherence to recommended COVID-19 vaccination. Factors such as a low education (13), being African American, and having a low household income were associated with higher COVID-19 vaccine hesitancy compared to being white and having a higher income (14). Large families were also a significant barrier to the intention to receive COVID-19 vaccination (15). Gaughan et al. (16) reported a lower COVID-19 vaccination rate among all minority communities compared to the white native population in England. A study conducted in Israel, before the COVID-19 vaccine became publicly available, found that both men and women in the Arab population had less intention to be vaccinated against COVID-19 compared to the Jewish population. Specifically, Arab men were 0.26 more times likely (95%CI = 0.16–0.42) to refuse COVID-19 vaccination compared to Jewish men, and Arab women were 0.42 more times likely (95%CI = 0.31–0.56) to refuse the COVID-19 vaccine compared to Jewish women (17). Moreover, the Arab population is less compliant with vaccinations for seasonal flu compared to the Jewish population. Receiving the flu vaccination is part of a healthy life style and of utilizing preventive health services (18). Studies show an association between previous intake of the seasonal flu vaccine and adherence to COVID-19 vaccination (19). Nevertheless, other social factors (beyond SES variables) in the Arab population in Israel that may affect their adherence with COVID-19 vaccination, such as health behavior and perceptions or acculturation strategies, have not yet been examined.

Acculturation strategy was first described by the psychologist John W. Berry in 1997. Barry's acculturation model refers to an individual's reconciling strategy to acclimate to their society (20). The model has been studied among immigrants and a variety of ethnic minority populations (21–23). The acculturation model describes two dimensions in an individual's perception: (i) his/her identity with their native culture, and (ii) his/her identity with the dominant society culture. Four acculturation strategies (or styles) emerge from those two dimensions: *Integration*: The integration acculturation style occurs when the individual can identify with both his native culture and the dominant society culture. In this situation, there is a certain level of preservation of identity with the original heritage culture and at the same time the desire to participate in and be an integral part of the large social network of the dominant society. *Assimilation*: The assimilation acculturation style occurs when the individual tends to embrace and absorb the values of the dominant society in an attempt to resemble as much as possible the members of the dominant new society. In this situation individuals prefer to remove themselves from their heritage culture and assimilate among the dominant society. *Separation*: The separation acculturation style occurs when the individual tends to preserve the pattern of their original culture and prefers to avoid contact with members of the dominant culture. The individual is not involved in groups from the dominant culture and prefers to turn inward toward their heritage culture. *Marginalization*: The marginalization acculturation style occurs when the individual suffers from a sense of alienation and loss of identity from both their heritage culture and the dominant society culture. The individual loses their connection with their heritage culture but also fails to develop new connections with the dominant society (20, 24).

Studies have found acculturation style and health behavior to be associated but the findings are inconsistent. Martinez et al. (21) found that a separation acculturation style was associated with a high risk of sexual behavior among women of Latin descent. Another study reported that immigrant women from Africa in the USA who adopted an integration acculturation style were seven times more likely (CI = 1.54–28.91) to undergo cervical cancer screening compared to those with a separation style (25). A study from northeastern USA found that Korean immigrants with a higher American orientation (i.e., an assimilation style) were more likely to have healthier dietary behavior related to decreased body image discrepancy compared to immigrants with Korean orientation (i.e., a separation style) (26). Conversely, Gerend et al. (27) found no significant association between acculturation and knowledge and health beliefs regarding HPV vaccination (27). These examples therefore indicate that there is no consensus as to which acculturation style is the most effective in adopting health behavior or adherence to vaccination.

## The syndemics framework

The term “syndemics” originates from two Greek words: “synergy” meaning working together and “epidemic” meaning upon the people. The syndemics theory promotes a holistic view of the individual and the community from a social, political, and economic perspective, and considers psychological factors that interact with different illnesses, diseases, and epidemics (28). The syndemics theory was first described by Merrill Singer, a health anthropologist, in the 1990's (29).

The syndemics theory has three components: 1. The co-occurrence of two or more diseases or health conditions in a given vulnerable population; 2. A bidirectionality or interrelationship between the diseases or health conditions; 3. Social-cultural conditions including economic and political circumstances that promote this co-occurrence and the interactions between the diseases or health conditions (30, 31). The consequence of this process is an increased disease burden or increased adverse health conditions in the specific population or community (28, 30). In other words, the syndemics theory argues that the co-occurrence of two diseases or health conditions are exacerbated in vulnerable populations or communities in the context of social, cultural, economic, and political circumstances. Namely, the existence of co-occurring illnesses or health conditions and the sociocultural context leads to the worst health outcomes in vulnerable communities. This means that people's sociocultural, political, and environmental context are essential factors for understanding the development of health status outcomes. Usually, this is most likely to emerge in conditions of health inequality caused by poverty, stigmatization, stress, ethnic inequality, immigration, or structural violence. These factors are associated with disease clustering and exposure and with increased physical and behavioral vulnerability (30, 32). Therefore, syndemics may explain the health disparities between different people or communities (33).The current study focused on two social health conditions among the Arab ethnic minority in Israel who display lower adherence to COVID-19 vaccination compared to the general Jewish population. The first was a syndemic construct which included three components: self-rated health status, health behavior, and whether they received the flu vaccine in the previous year. The second was their acculturation style. The aim of the study was to explore the association between acculturation style, syndemic construct, and adherence to the recommended COVID-19 vaccination in the Arab ethnicity minority in Israel.

## Materials and methods

### Study design and setting

A cross-sectional study was conducted during the second week of April 2021. At that time Israel had already undergone a third shutdown, with a COVID-19 incidence rate of 8-10/10,000 citizens. Worldwide, Israel was ranked first, with 55% of the population having been vaccinated with two doses of the COVID-19 vaccine (6).

Participants were recruited for the study through an internet polling service, *i-panel* (https://www.ipanel.co.il/en/) that matched the population in Israel (34) and was used in a variety of studies (35). The *i-panel* is a member of the European Society for Opinion and Marketing Research (ESOMAR). The eligibility criteria were adults of Arab ethnicity who could read Hebrew or Arabic. The questionnaire was distributed randomly to eligible members of the panel by email or cell phone message. The participants received a small reward from the internet polling service for participating in the study (a bookshop gift card).

The sample size was calculated using G^*^Power software (version 3.1.9.7) (36) for multiple linear regression with the following parameters: effect size *f*^2^ of small to medium of 0.09, alpha error of 5%, power of 80%, with 15 predictors. The total calculated sample size was 222 participants. I assumed that 15% of the participants had already been ill with COVID-19 and that there could be an attrition rate of 10% of the participants. Indeed, the initial study sample included 305 Arab participants but 43 reported previous COVID-19 (14.1%) infection and were therefore not candidates for vaccination. Hence, they were excluded from the final analysis and the final sample included 262 participants which satisfied the statistical power requirement.

### Tools and variables

Data were collected using a closed self-report questionnaire with five sections as follows:

#### Personal details

These included the age, gender, religiosity, family status, education level, and income.

#### Acculturation style

The participants were asked two questions according to Dona et al. (37) acculturation measurement that was adapted to the Israeli ethnic situation. The first question asked the participants to score their identity in belonging to the Israeli culture and the second question asked the participant to score their identity in belonging to the Arab culture. The score was on a 1 to 5 Likert scale where 1 was “an extremely strong sense of belonging” and 5 was “an extremely weak sense of belonging”. In the analysis phase, scores 1 and 2 were aggregated to a “yes” answer (= 1) and scores 3 through 5 were aggregated to a “no” answer (= 0) (22, 37). Subsequently, each participant could have one of the following four acculturation strategy scores as follows (for further explanation see Supplemental material 1): *Marginalization*: Score 0, if the answer to both questions was “no”. *Integration*: Score 2, if the answer to both questions was “yes”. *Assimilation*: Score 1, if the answer to the first question was “yes” and to the second question was “no”. *Separation*: Score 1, if the answer to the second question was “yes” and to the first question was “no”. In the current study, a low correlation was found between the two questions (*r* = 0.19, *p* < 0.01), suggesting that high affinity with the Israeli culture is not necessarily associated with low affinity with the Arab culture (and vice versa). In fact, there is a continuum between the two dimensions (22).

#### Health behavior index

This questionnaire was adapted from the Israeli Knowledge, Attitude, Practice (KAP) survey. The survey collects information regarding the lifestyle of the Israeli population (38). The current study used six questions from the KAP survey that deal with health behavior. For example, the participants were asked if they smoke cigarettes with possible responses of “do not smoke”, “smoked in the past”, “sometimes smoke”, and “smoke every day”. Other questions dealt with drink sugary drinks, exercise, family meal habits, consumption of processed and industrialized foods, and consumption of whole meal bread. The scores for each question were between 1 and 4. The higher the score, the higher the risk behavior. The total score on the six questions comprised the health behavior index.

#### Adherence to the recommended vaccinations

The participants were asked if they had been vaccinated against COVID-19 and flu vaccine. The following responses were scored 1–5 respectively: “I have no intention of being vaccinated”, “I probably won't be vaccinated”, “I'll probably be vaccinated”, “I intend to vaccinate”, and “I have already been vaccinated”.

#### Self-rated health status

The participants were asked a general question on how they subjectively scored their health status on a 1–5 Likert scale where 1 = “excellent” and 5 = “poor”. This single general health measure was found to have good reliability across gender and age (39) and to be a good predictor for health behavior, morbidity and mortality (40–42). The higher the score, the worse the reported health status.

### Statistical analysis

Descriptive statistics was used to describe the sample characteristics and variables. Associations between the syndemics score (explained below) and the sample characteristics were examined with chi-square tests. A normal distribution of the variables was checked with VIF <1.07; tolerance >0.10; normal probability plot; Durbin-Watson statistic = 1.966. Analysis of all these parameters suggested that the variables had a normal distribution (43).

A syndemic construct was calculated with two dimensions: syndemics score and syndemics severity as suggested by Martinez et al. (21). Three independent variables were used to calculate the syndemics score and the syndemics severity. The variables were: health behavior index (including smoking, drinking sugary drinks, exercise, family meal habit, consumption of processed and industrialized foods, and eating whole meal bread), self-rated health status, and receiving the flu vaccine. These variables were recoded into dichotomized 1/0 scores. For example, for the self-rated health status “very good” to “excellent” answers were recoded to score “0” and “good” to “poor” answers were recoded to score “1” (see Supplemental material 2). From the above three measures, the syndemics score and syndemics severity score were calculated as follows:

#### Syndemics score

The three variables of health behavior index, self-rated health status, and receiving the flu vaccine were scored between 0 and 3 where 0 indicated no reported risk factors and 3 indicated that the participant reported all three risk factors (high risk health behavior index, bad self-rated health status, and no flu vaccine received). The higher the score, the higher the number of risk factors, and the higher the syndemics score. The health behavior index was calculated as follows: The variables of smoking, drinking sugary drinks, exercise, family meal habit, consumption of processed and industrialized foods, and eating whole meal bread were recoded to dichotomous yes/no answers (1/0, respectively). For example, the answers for the smoking question of “do not smoke” and “smoked in the past” were recoded to “no” (= 0) while the “sometimes smoke” and “smoke every day” were recoded to “yes” (= 1). Notably, for the question of whether the participants eat whole meal bread, the coding was reversed since eating whole meal bread is considered a good health behavior. Therefore, answers of “yes, for the most part” and “yes, always” were recoded to “no” (= 0) and “sometimes” and “not at all” were recoded to “yes” (= 1). The score “1” therefore indicated a risky health behavior. Accordingly, the minimum total score for health behavior was “0” which indicated that the participant had no risky health behavior while the maximum sum score of “6” indicated that the participant had all six risky health behaviors. Lastly, the health behavior index was recoded to a dichotomous variable. Scores “0” to “1” were combined to “0” meaning the participant had no risky health behavior and scores “2” to “6” were combined to “1” meaning the participant had highly risky health behavior.

#### Syndemics severity score

This score indicated the severity of the risk factors reported by the participants. Health behavior, self-rated health status, and adherence/receiving the flu vaccine were all scored 1–4. These values were summed (with a score range of 4–12) and the higher the score, the higher the severity of the risk factors, and the higher the syndemics severity.

A linear regression with the stepwise method was conducted to examine the effect of the explanation variables on the dependent variable. The explanation variables were the personal details, acculturation style, and syndemic construct of the participants. The dependent variable was their adherence to the COVID-19 vaccine. The explained variance (*R*^2^) was calculated. The statistical analysis used SPSS software version 27 and *p* < 0.05 was considered to be statistically significant.

### Ethical consideration

The study was approved by the ethical committee of the Tel Aviv University (#0002629-1). At the beginning of the questionnaire there was short explanation of the study and a request to apply for participation in the study. Participants were assured complete anonymity and that they could retire from filling out the questionnaire at any time they wished.

## Results

The research sample's characteristics are described in Table 1. Most of the participants were female (56.1%), with an academic education level (57.3%). The mean age was 32.8 (SD = 11.21). Most of the participants were classified with separation acculturation style (43.5%) and the second most common acculturation style was integration (28.6%). The sample's syndemics scores are presented in Table 2. Most of the participants had a syndemics score of 2 (40.8%) indicating two risk factors. Only 5.4% have syndemics score of zero. A Chi-square test for categorical variables and a one-way ANOVA for continuous variables (e.g., age) revealed no statistically significant associations between the participants' syndemics scores (0, 1, 2, 3) and their personal information. An integration acculturation style was significantly associated with syndemics score. Namely, participants with an integration style had higher syndemics scores of 2 and 3 compared to participants with marginalization and separation styles (45.3 vs. 43.3%, 37.7 and 22.7 vs. 16.7%, 7.9%, *p* = 0.01, respectively).

**Table 1:** Sample characteristics (*n* = 262).

**Variable**	***N*** **(%)**
**Gender**	
Male	115 (43.9) 147
Female	(56.1)
**Marital status**	
Married	144 (55.0) 118
Single	(45.0)
**Religiosity**	
Secular	73 (27.9) 189
Traditional-orthodox religion	(72.1)
**Education**	
Elementary	5 (1.9)
High school	72 (27.5)
Diploma	35 (13.4)
University	150 (57.3)
**Income**	
Below average	205 (78.2)
Average	44 (16.8)
Above average	13 (5.0)
**Had relative who was ill with COVID-19**	
Yes	146 (55.7)
No	116 (44.3)
**Been in isolation**	
Yes	84 (32.1)
No	178 (67.9)
**Acculturation style**	
Marginalization	60 (22.9)
Separation	114 (43.5)
Integration	75 (28.6)
Assimilation	13 (5.0)
Age (M and SD)	32.8 (11.21)

**Table 2:** Sample characteristics by syndemics score (*n* = 262).

**Variable**	**Score 0**	**Score 1**	**Score 2**	**Score 3**	**χ^2^**	***p*** **=**
	**N (%)**	**N (%)**	**N (%)**	**N (%)**		
Total	14 (5.34)	94 (35.87)	107 (40.83)	37 (14.12)		
Gender					0.48	0.92
Male Female	10 (8.7) 14 (9.5)	39 (33.9) 55 (37.4)	49 (42.6) 58 (39.5)	17 (14.8) 20 (13.6)
Marital status					3.35	0.34
Married Single	13 (9.0) 11 (9.3)	45 (31.3) 49 (41.5)	63 (43.8) 44 (37.3)	23 (16.0) 14 (11.9)
Religiosity					0.69	0.87
Secular Traditional-orthodox religion	8 (11.0) 16 (8.5)	24 (32.9) 70 (37.0)	31 (42.5) 76 (40.2)	10 (13.7) 27 (14.3)
Education	10 (8.9)				1.90	0.59
School University	14 (9.3)	36 (32.1) 58 (38.7)	51 (45.5) 56 (37.3)	15 (13.4) 22 (14.7)
Income					4.82	0.18
Below average Average-above average	15 (7.3) 9 (15.8)	72 (35.1) 22 (38.6)	88 (42.9) 19 (33.3)	30 (14.6) 7 (12.3)
Had relative who was ill					2.46	0.48
with COVID-19 Yes	15 (10.3) 9 (7.8)	57 (39.0) 37 (31.9)	55 (37.7) 52 (44.8)	19 (13.0) 18 (15.5)
NO						
Was in isolation					7.42	0.06
Yes No	12 (14.3) 12 (6.7)	35 (41.7) 59 (33.1)	28 (33.3) 79 (44.4)	9 (10.7) 28 (15.7)
Acculturation style^a^					15.91	0.01
Marginalization Separation Integration	4 (6.7) 15 (13.2) 2 (2.7)	20 (33.3) 47 (41.2) 22 (29.3)	26 (43.3) 43 (37.7) 34 (45.3)	10 (16.7) 9 (7.9) 17 (22.7)
	M (SD)	M (SD)	M (SD)	M (SD)	F	*p* =
Age^+^	35.3 (14.17)	32.1 (10.13)	32.3 (11.31)	33.8 (11.21)	0.89	0.44

a The assimilation acculturation style was not analyzed because of small number of participants in this group. Therefore *n* = 249 here.

+ One-way ANOVA.

The linear regression (stepwise method) revealed that older age, men, and not being in previous isolation were statistically significant associated with higher adherence to recommended COVID-19 vaccination (*B* = 0.03, 95%CI = 0.02–0.5; *B* = 0.45, 95%CI = −0.78–0.11; *B* = −0.51, 95%CI = −0.87–0.16, respectively). Moreover, both assimilation and separation acculturation styles were statistically significant associated with higher adherence to recommended COVID-19 vaccination (*B* = 1.12, 95%CI = 0.34–1.98; *B* = 0.45, 95%CI = 0.10–0.80; respectively). Finally, syndemics severity was statistically significantly associated with higher adherence to the recommended COVID-19 vaccination (B = 0.18, 95%CI = 0.09–0.28). This last finding indicated that participants with a higher severity of health behavior who did not receive the flu vaccine and had lower self-health rated status, had more adherence to the recommended COVID-19 vaccination. Notably, the syndemics scores were not associated with adherence to COVID-19 vaccination. The explained variance of the model (*R*^2^) was 19.9% (Table 3)

**Table 3:** Linear regression^a^ (stepwise method) to explain adherence to recommended COVID-19 vaccination.

**Variable^b^**	**B**	***p***	**95%CI**
Age	0.03	<0.001	0.02;0.5
Gender	−0.45	0.009	−0.78; −0.11
Having been in isolation	−0.51	0.005	−0.87; −0.16
Assimilation acculturation	1.12	0.005	0.34;1.98
Separation acculturation	0.45	0.01	0.10;0.80
Syndemics severity	0.18	<0.001	0.09;0.28

a Linear regression with stepwise method conducted with six steps. *R*^2^ = 19.9% and Δ*F* = 0.01.

b The variables: religiousness, family status, education level, income, one of the family members have been ill with COVID-19. Integration and marginalization acculturation styles were not statistically significant as explanation variables.

## Discussion

The current study aimed to explore the association between acculturation style, syndemic construct and adherence to the recommended COVID-19 vaccination in the Arab ethnicity minority in Israel. The findings suggested that most of the participants had one to three syndemics risk factors. Nevertheless, a linear regression revealed that the participants' syndemics severity explained their adherence to the recommended COVID-19 vaccination. These findings emphasize that the syndemics severity of lifestyle risk factors may affect adherence to COVID-19 vaccination and not merely the number of the syndemics factors. The importance of syndemics severity was found in a previous study regarding healthy sexual behavior among young Latina women (21). There was a paradoxical finding in the current study; participants with higher syndemics severity had the most adherence to COVID-19 vaccination. From a practical perspective, this is a desirable situation and healthcare professionals have advised people with high risk factors (such as smoking) to receive the vaccine. Indeed, the mass COVID-19 vaccination campaign initially prioritized giving the vaccine to older people and those with medical risk factors (6). However, higher risk factors (e.g., not exercising and smoking) may be associated with chronic disease, as well as higher morbidity and mortality (44). Accordingly, it may be suggested that participants with higher syndemics severity perceive themselves as less healthy and feel less health security. These feelings therefore may motivate them to receive the COVID-19 vaccine. In contrast, participants with lower syndemics severity scores had less adherence to vaccination which may make them more vulnerable to morbidity and mortality from COVID-19. In terms of public health, this is a complex challenge. The findings of the study therefore reinforce the need to target certain audiences during a COVID-19 vaccination campaign (45). Specifically, the COVID-19 vaccine campaign should be targeting population groups with both higher and lower syndemics severity but with differential health messages that are tailored to the particular health behavior. At the same time, it is important that the health message regarding COVID-19 vaccination is culturally tailored (16) to the Arab minority in Israel. Studies from Israel have found that the Arab minority do less exercise, smoke more (among the males), have less adherence to dietary recommendations, and have less adherence to seasonal flu vaccination compared to the general population (38, 46). The Arab minority also rate their health status lower than the Jewish population (47). This translates into higher rates of morbidity and mortality in the Arab minority compared to the Jewish majority (46, 48). Also, the Israeli Arab minority have lower SES compared to the Israeli Jewish population (7). Studies worldwide shows that low SES populations suffer from unhealthy lifestyles compared to high SES populations (49). On the other hand, health education and health promotion programs among the lower SES population may encourage healthy lifestyle behaviors such as smoking cessation (50), undergoing medical screening tests (51), seasonal flu vaccination uptake (52), and healthy nutrition (53). Such health programs can also encourage personal and community empowerment (47). In terms of promoting public health, efforts should be made to encourage a healthy lifestyle among all sectors of the population regardless of the rates of COVID-19 vaccination.

Assimilation and separation acculturation styles were found in this study to be associated with adherence to the recommended COVID-19 vaccination among the Arab ethnic group. As far as is known, few studies have been conducted to assess the association between acculturation and vaccination and of these, most have examined the association between acculturation and human papillomavirus vaccine (HPV). In the latter studies, acculturation was measured by the parameters of being native borne, years of living in the host country, or ability to speak in the native host language rather than acculturation style (15, 54). a study from USA found that students with Asian orientation (affinity to separation style) were less likely to have the HPV vaccine and less likely to consult with health professionals regarding the HPV vaccine compared to students with Western orientation (affinity to assimilation style) (55). Another study found that an assimilation acculturation style was associated with stress among young Muslim ethnic groups in Britain while a separation acculturation style was associated with stress in interactions with other minority groups (23). There seems to be a contradiction between the separation and assimilation acculturation style. Nguyen and Benet-Martínez (2013) argued that the outcome of acculturation depends on multidomain variables and the way the acculturation is measured and acculturation is a complex and multidimensional process. For example, people may choose one acculturation style in their home and another acculturation style in their work or social life. Indeed, individuals' acculturation style, health behavior, and health outcomes are associated with their life circumstances, practices, and values (56). Berry and Hou (2016) found that both assimilation and separation acculturation styles have benefits for immigrant well-being (regarding life satisfaction and mental health). A study of Latina/o parents found that higher assimilation orientation and higher separation orientation interact to predict positive beliefs and self-efficacy regarding HPV vaccination (27). In the current study, the assimilation style may reflect the desire to adopt the Jewish behavior of higher vaccination against COVID-19 than among Arab society (6, 8). It is possible that the positive association between the separation style and COVID-19 vaccination is based on the positive perception and commitment of the Arab population to the national childhood immunization program, with its higher vaccination rate compared to the Jewish population (57). Nonetheless, there are studies that indicated a low COVID-19 vaccination coverage rate among Arab society, linked to low credibility of the government's recommendations for COVID-19 vaccination (17) and issues of inequality (58). In light of this, it seems that the comprehensive pattern of acculturation has an impact on health outcomes. Therefore, to encourage COVID-19 vaccination among the Arab ethnicity, health policymakers need to identify, alongside acculturation style, the sources that support, reinforce and encourage the specific acculturation style and tailor the vaccination campaign accordingly. Thus, the health message that encourages vaccination should be tailored according to the socio-cultural nature of these acculturation styles. For example, by emphasizing the benefits of the vaccine within heritage family values (separation style) along with the benefits of the vaccine within social values (assimilation style). At the same time, it is important to find health messages that encourage COVID-19 vaccination for the acculturation styles that have not been found to be associated with vaccination (i.e., integration and marginalization styles) rather than change the acculturation style.

## Limitations

The current study is an exploratory study and presents new aspects regarding adherence to COVID-19 vaccination but it has several limitations. First, it was an internet study using a polling panel which means no probability sampling. People who were not included in the panel could not participant in the current study. Moreover, the participants in the current study do not necessarily represent Israel's entire Arab population. For example, the mean age of the participants was 32.8 (SD = 11.2), meaning low representativeness of older people. Second, the syndemics structure included three determinants of health (health behavior index, self-rated health status, and receiving the seasonal flu vaccine). It could be that choosing other health determinants would impact COVID-19 vaccination differently. Finally, the acculturation style was examined in one way (Barry's acculturation style) but there are several ways to examine acculturation among minority communities that were not used in the current study.

## Conclusion

Syndemics severity and assimilation and separation acculturation styles were associated with higher adherence to COVID-19 vaccination in the Israeli Arab minority population. The syndemics severity is a meaningful factor rather than being simply the number of risk factors (the syndemics score). The health system should encourage a healthy lifestyle and at the same time promote COVID-19 vaccination for both higher and lower syndemics severity populations. Moreover, a campaign to encourage COVID-19 vaccination among minority populations should be tailored to the social acculturation style in a sensitive and individualized manner. Future research should be conducted regarding the impact of syndemics structure, acculturation, and COVID-19 vaccination among minority and vulnerable populations in other countries.

## Data availability statement

The data analyzed in this study is subject to the following licenses/restrictions: The data presented in this study are available upon reasonable request from the corresponding author. Requests to access these datasets should be directed to: AAA anatamit@tauex.tau.ac.il.

## Ethics statement

The studies involving human participants were reviewed and approved by Tel-Aviv university Ethical Committee (# 0002629-1 dated 16.1.2021). The patients/participants provided their written informed consent to participate in this study.

## Author contributions

AA: Conceptualization, methodology, writing original draft, validation, formal analysis, investigation data curation, visualization, editing.

## Funding

This research did not receive any specific grant from funding agencies in the public, commercial, or not-for-profit sectors.

## Conflict of interest

The author declares that the research was conducted in the absence of any commercial or financial relationships that could be construed as a potential conflict of interest. The handling editor BA declared a shared affiliation with the author at the time of review.

## Publisher's note

All claims expressed in this article are solely those of the authors and do not necessarily represent those of their affiliated organizations, or those of the publisher, the editors and the reviewers. Any product that may be evaluated in this article, or claim that may be made by its manufacturer, is not guaranteed or endorsed by the publisher.
