# Supplementary material for: Social determinants and adherence to recommended COVID-19 vaccination among the Arab ethnic minority: A syndemics framework
Source: Front Public Health. 2022 Sep 28;10:1016372. doi: 10.3389/fpubh.2022.1016372 (PMC9554497; doi:10.3389/fpubh.2022.1016372)
Supplement: Supplementary file 1 [file Data_Sheet_1.docx]

**Supplemental material 1:** Scoring of acculturation style

|  | | | 1 Extremely strong | 2  Strong | | 3  Slightly strong | 4  Slightly weak | 5  Extremely weak | |
| --- | --- | --- | --- | --- | --- | --- | --- | --- | --- |
|  |  |  | Yes=1 | | | No=0 | | | |
| To what extent do you identify with the Israeli culture? | Yes=1 | Integration  (score=2) | | | Assimilation  (score=1) | | | |  |
| To what extent do you identify with the Arab culture? | No=0 | Separation  (score=1) | | | Marginalization  (score=0) | | | |  |

Source:

Dona G, & Berry J.W. (1994). Acculturation Attitudes and Acculturative Stress of Central American Refugee. *International Journal of Psycology, 29*(1), 57-70

**Supplemental material 2**: Classification of the independent variables for the syndemics construct

| Independent variables | | | Recoding for syndemics construct | |
| --- | --- | --- | --- | --- |
| Variable | Definition | Original score | Syndemics score | Syndemics severity |
| Health behavior index | Continuous variable including: Smoking, regular exercise, healthy nutrition. | 1-4 | 0/1:  0= No risk factor  1= Risk factor | 1-4 |
| Self-rated health status | Continuous variable | 1-5 | 0/1:  0=high self-rated health  1= low self-rated health | 1-4 |
| Received a flu vaccine in the last year | Continuous variable | 1-4 | 0/1:  0=received vaccine  1= did not receive vaccine | 1-4 |
